# Late Deprescribing of Angiotensin-Converting-Enzyme Inhibitors and Renin-Angiotensin Blockers in Patients with Advanced Cancer Receiving Palliative Care

**DOI:** 10.1177/26892820251372015

**Published:** 2025-08-28

**Authors:** Linda Björkhem-Bergman, Christel Hedman, Máté Szilcz, Gabriella Frisk

**Affiliations:** ^1^ Department of Neurobiology, Division of Clinical Geriatrics, Care Sciences and Society (NVS), Karolinska Institutet, Huddinge, Sweden.; ^2^ Department/Palliative Care, Stockholms Sjukhems R&D, Stockholm, Sweden.; ^3^ Department of Molecular Medicine and Surgery, Karolinska Institutet, Stockholm, Sweden.; ^4^ Department of Clinical Sciences Lund, Lund University, Lund, Sweden.; ^5^ Department of Medical Epidemiology and Biostatistics, C8 Medicinsk epidemiologi och biostatistik, MEB Jonell, Karolinska Institutet, Stockholm, Sweden.; ^6^ ASIH Stockholm Södra, Advanced home care and Specialized palliative ward, Nacka, Sweden.

**Keywords:** antihypertensivs, deprescribing, pharmacology

## Abstract

**Background:**

Treatment with antihypertensives in patients with advanced cancer is often continued until very late in the disease trajectory, despite a considerable risk of hypotension.

**Objectives:**

The aim of this study was to investigate the time of deprescribing of antihypertensive agents in patients with cancer receiving palliative care during their last year of life. The monitoring of blood pressure (BP) during treatment was also studied.

**Design:**

Retrospective cohort study.

**Setting/Subjects:**

Medical records of all patients admitted during a three-year period to a home care unit in Stockholm, Sweden, and now deceased were screened for antihypertensive agents. To create a homogenous cohort, only agents of the renin-angiotensin system (ATC-code C09) were included.

**Measurements:**

Data for time of deprescribing and monitoring of BP were collected.

**Results:**

Of 1501 deceased patients, 353 had been treated with agents of the renin-angiotensin system for hypertension and had a primary diagnosis of cancer. BP was measured before deprescribing in 169 patients (47.9%). In 102 patients (28.9%), antihypertensive treatment continued up to the last seven days of life. For 27 patients (7.6%), the treatment had not been deprescribed. In 184 patients (52, 1%), BP was not followed up despite continued antihypertensive treatment. All 27 patients whose treatment was never deprescribed were in this group.

**Conclusions:**

This study shows that antihypertensive treatment is often deprescribed late or not at all in patients with advanced cancer. Monitoring BP in patients treated with antihypertensives in palliative care may facilitate making the decision to deprescribe them in time.

## Introduction

In advanced stages of cancer, when patients are approaching the end of life, the primary focus of care often shifts from curative treatment to optimizing quality of life (QoL), where the management of symptoms becomes essential.

Medications aimed at primary or secondary prevention should not be prioritized when the prognosis is poor and life expectancy is from weeks to months.^1,2^ However, these medications are often deprescribed very late, even in the final days of life. Antihypertensive drugs, often prescribed for long-term cardiovascular health, may not only lose their relevance but might also cause side effects and unnecessary complications.^3–5^

Previous research indicates that many patients with advanced cancer experience fluctuating and low blood pressure (BP) caused by cachexia, weight loss, dehydration, reduced physical activity, and the systemic effects of the disease.^6^ In such cases, the continuation of antihypertensive medications may cause adverse outcomes, such as increased fatigue, dizziness, and falls, which can reduce the patient’s overall QoL.^6,7^

Deprescribing is the process of identifying and discontinuing medications with no or minimal benefit or with the potential for harm, to align with established goals of care and to reduce tablet burden.^8^ However, several barriers to deprescribing have been described.^2,9^ The prescription of medication is often driven by guidelines, but recommendations regarding discontinuation are generally lacking for most conditions.

The aim of this study was to investigate when antihypertensive drugs, specifically agents acting on the renin-angiotensin system (ATC code C09), were deprescribed in relation to time of death, and how BP was measured prior to deprescribing. The reason for choosing to only investigate this group of antihypertensive medications was to create a homogeneous cohort and reduce variability and confounding factors. This group of antihypertensive medications is also commonly used in Sweden and easy to deprescribe without scaling down.

## Methods

### Study design

This is a retrospective single-center cohort study. Medical records of all patients admitted to a specialized home care unit in Stockholm, Sweden,” ASIH Stockholm Södra,”^10,11^ during a three-year period (January 1, 2016, until December 31, 2018) were screened.

The study was approved by the Swedish Ethical Review Board, Dnr 2018/1798-31. No informed consent was obtained since all patients included were deceased at the time of the retrospective review.

### Study settings

The unit provides specialized home care and a specialized palliative ward for patients and enrolls about 400 outpatients and 16 inpatients at any given time. The unit is staffed by specialized palliative care physicians and physicians in training. In the specialized palliative home care, 80% of patients have malignancies and 20% have nonmalignant diseases. The average duration of care is three months for the specialized home care and one to two weeks for the specialized palliative ward. Since all health care units in Region Stockholm have joint medical records, we could collect data on prescribed drugs for all patients during their last year of life, regardless of place of care. The patients could also move between the home care teams and the in-patient ward during their enrollment in the palliative care unit. When patients are admitted for specialized palliative care, the unit has the overall responsibility for the patients; the patients do not, therefore, have contact with their general practitioner during this time. The medical regimens are reviewed with the patients, and, apart from antitumoral treatments, all prescriptions are made by the specialized palliative care physicians.

### Participants

The medical records of all patients who had been admitted to the palliative care unit over the three-year study period and were now deceased were screened. Patients were included if they had a primary cancer diagnosis and medication prescribed during the last year of life acting on the renin-angiotensin system, that is, “angiotensin-converting-enzyme (ACE)-inhibitors” and/or “angiotensin-II-receptor-blockers” (ARB) (ATC code C09), where the indication was high BP. Patients who had been prescribed these medications for heart failure were excluded.

### Variables

The following data were extracted from the patients’ medical records: sex, age, primary diagnosis, time and cause of death, type of medication and dose of antihypertensives (ATC-code C09), date of deprescribing, complications after deprescribing (stroke or myocardial infarction), and BP at time of admission to palliative care and at time of deprescribing antihypertensives.

### Statistical methods

Descriptive statistics are presented as *n* (number) and percentage (%). The chi-square test was used for statistical analysis of categorized variables, and an unpaired *t*-test was used for continuous variables. We employed a proportional odds regression model to examine the association between gender and time of deprescribing, adjusted for age and systolic BP at the time of admission. To account for the ordinal nature of the time of deprescribing variable, we used a cumulative link model with a logit link to estimate the odds of being in an earlier deprescribing category based on gender while controlling for age (continuous) and systolic BP (continuous) at admission. Since the systolic BP variable had missing values, we applied multiple imputations using predictive mean matching to generate five imputed datasets, which were then combined in the final analysis to reduce potential bias from missing systolic BP data.

The statistical analyses and figures were made in R version 4.3.1, Excel, and Graph Pad Prism, versus 9.5.0.

## Results

Of 1501 deceased patients with cancer, 353 had been treated with ACE-inhibitors or ARB for hypertension. The majority were men (*n* = 202, 57.2%) and the mean age at time of death was 74.3 years, range 44–99 years. Regarding type of medication, 166 (47.0%) patients had received ACE-inhibitors, 14 (4.0%) ACE-inhibitors in combination with diuretics, 126 (35.7%) ARB, and 47 (13.3%) ARB in combination with diuretics. Statistically significantly more women had received treatment with ARB (*p* = 0.029) (Table 1).

**Table 1.** Characteristics of 353 Patients with Palliative Cancer Diagnoses and Hypertension, and Treatment with Medication Acting on the Renin-Angiotensin System (ATC-Code C09) in the Last Year of Life

	Patients *N* (%)	Men *N* (%)	Women *N* (%)	*p* value^*^
Total	353 (100)	202 (100)	151 (100)	
Age at time of death				
Mean age 74.3 years				
<65	56 (15.9)	33 (16.3)	23 (15.2)	n.s.
65–80	192 (54.4)	115 (56.9)	77 (51.0)	n.s.
>80	105 (29.7)	54 (26.7)	51 (33.8)	n.s.
Level of care in the unit				
Specialized palliative home care	256 (72.5)	156 (77.2)	100 (66.2)	n.s.
Specialized palliative ward	97 (27.5)	46 (22.8)	51 (33.8)	n.s.
Diagnosis				
Brain tumors	11 (3.1)	6 (3.0)	5 (3.3)	
Breast cancer	17 (4.8)	1 (0.5)	16 (10.6)	
Gastrointestinal cancer	140 (39.7)	86 (42.6)	54 (35.8)	
Gynecological cancer	17 (4.8)	0 (0.0)	17 (11.3)	
Head and neck cancer	8 (2.3)	4 (2.0)	4 (2.6)	
Hematological malignancies	16 (4.5)	8 (4.0)	8 (5.3)	
Lung cancer	62 (17.6)	34 (16.8)	28 (18.5)	
Urological cancer	50 (14.2)	44 (21.8)	6 (4.0)	
Other cancers	32 (9.1)	19 (9.4)	13 (8.6)	
Type of medication				
ACE-inhibitors	166 (47.0)	106 (52.5)	60 (39.7)	n.s.
ACE-inhibitors combination	14 (4.0)	8 (4.0)	6 (4.0)	n.s.
ARBs	126 (35.7)	60 (29.7)	66 (43.7)	**0.029**
ARBs combination	47 (13.3)	28 (13.9)	19 (12.6)	n.s.
Time of deprescribing (days before death)				
Not deprescribed	27 (7.6)	16 (7.9)	11 (7.3)	n.s.
Same day as death	2 (0.6)	2 (1.0)	0 (0.0)	n.s.
1–3 days before	47 (13.3)	26 (12.9)	21 (13.9)	n.s.
4–7 days before	26 (7.4)	11 (5.4)	15 (9.9)	n.s.
8–14 days	32 (9.1)	18 (8.9)	14 (9.3)	n.s.
15–28 days	42 (11.9)	28 (13.9)	14 (9.3)	n.s.
29–180	132 (37.4)	69 (34.2)	63 (41.7)	n.s.
>181	45 (12.7)	32 (15.8)	13 (8.6)	n.s.

Bold values are statistically significant, *p* < 0.05.

^*^ Statistical analysis was performed using chi-square-test, *p* < 0.05 was considered statistically significant.

n.s., not significant.

### Deprescribing

**FIG. 1.**
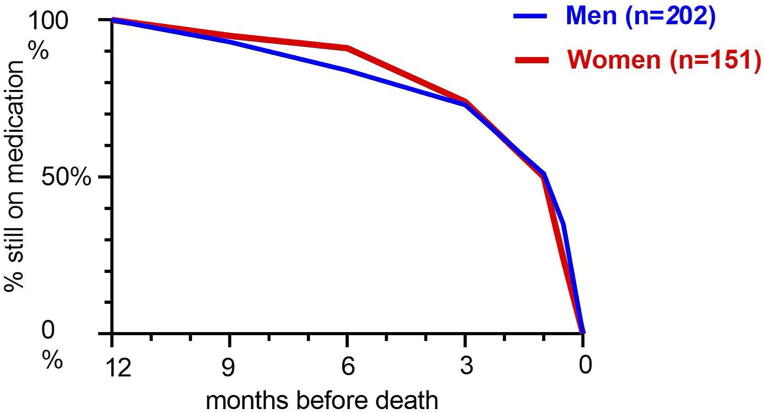
Time of deprescribing of antihypertensives acting on the renin-angiotensin system (ATC-code C09) in women and men the last year of life.

For 102 patients (28.9%), the treatment continued up to the last seven days of life, and for 27 patients (7.6%), the treatment was not deprescribed at all. In 184 patients (52, 1%), BP was not followed up despite continued treatment. All 27 patients whose medication was never deprescribed were in this group. There was no significant difference in time of deprescribing between men and women (*p* = 0.59) (Table 1, Fig. 1). This result remained the same in the proportional odds regression model adjusted for age and systolic BP.

Four patients (1.2% of the deprescribed patients) suffered a myocardial infarction or stroke after deprescribing. According to the medical records, no BP had been monitored in these patients before deprescribing.

### Blood pressure

**FIG. 2.**
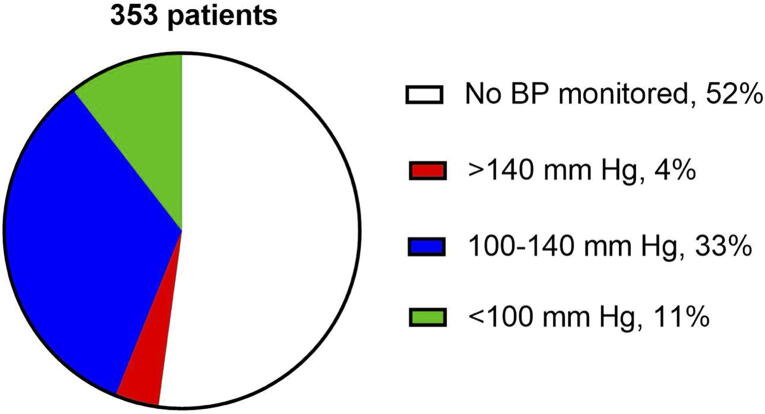
Systolic blood pressure (BP) at time of deprescribing in 353 patients treated with antihypertensives acting on the renin-angiotensin system (ATC-code C09) in palliative care.

BP was measured before deprescribing in 169 patients (47.9%) but not measured in 182 (52.1%) patients (Fig. 2). Of the patients whose BP had been measured before deprescribing, 14 (4.2%) had a systolic BP >140 mm Hg, 118 (33.4%) had between 100 and 140 mm Hg, and 37 (10.5%) had less than 100 mg Hg (Fig. 2).

## Discussion

This study shows that deprescription of ACE-inhibitors and ARB for hypertension among patients with cancer occurs late in the disease trajectory. Almost a third of the patients continued with antihypertension treatment until the last week of life, despite being enrolled in specialized palliative care. The study further indicates that BP is not measured adequately to detect changes in BP and therefore deprescribe medications in time. This may lead to a risk for hypotension, which can radically worsen the patient’s overall QoL. Moreover, the tablet burden might affect well-being during the end of life.

In the present study, only 4% of the patients had a systolic BP >140 mmHg, which may be considered a level indicating treatment to avoid cardiovascular complications in the long-term.^12,13^ However, in the current cohort, BP >140 might not have been an indication for antihypertensives since the life expectancy of patients was short. The overwhelming majority of patients had no measured or low BP, indicating potential overtreatment.

Our results are in line with other studies, such as a British study that showed that 25 of 26 study participants from a hospice were considered to have their antihypertensive medication inappropriately prescribed according to their current BP and remaining life expectancy.^3^

A study from the U.S. (*n* = 45) reported that 69% of the antihypertensive drugs given to patients admitted to palliative care should have been deprescribed,^14^ indicating a high level of overtreatment.

Deprescribing antihypertensives can, however, be controversial for both the clinical staff and the patient, since they are informed about how important these medications are for preventing cardiovascular diseases.^15^ As the recommended levels for prescribing antihypertensive treatments have been further tightened to <130/80, or if not tolerated <140/80,^12,13^ it can now be more challenging to know when during the disease trajectory it is appropriate to discontinue medication.

Deprescribing at the end of life can be challenging, as patients and health care professionals often hold differing views on what constitutes the best course of action. While clinicians may prioritize comfort and QoL,^16^ patients or their families may place greater value on continuing long-standing treatments, even when the benefits are uncertain in a palliative context. These differing perspectives can complicate shared decision-making, especially when emotions, hope, or a lack of understanding about prognosis and treatment goals come into play. Navigating these conversations requires sensitivity, clear communication, and a strong therapeutic relationship to ensure that the care aligns with the patient’s values and evolving clinical needs.^17,18^

The study shows that there was no difference between men and women regarding the time of deprescribing. This is in contrast with our studies on statins, where women were deprescribed statins much earlier in the disease trajectory than men.^19,20^

Difficulty swallowing tablets typically arises during the final days of life, which might have been the reason for deprescribing. However, deprescribing at this stage is very late, and the medication might have been associated with serious side effects.

To conclude, the medical staff and the patients should participate in shared decision-making regarding prescribed medications, including when treatment should be intensified, tapered down, or discontinued when they can do more harm than good. Moreover, if BP is monitored in patients in palliative care who are treated with antihypertensives, it may facilitate the decision to deprescribe them earlier. Since deprescribing medications can be challenging, a future qualitative study could be valuable to explore why physicians perceive deprescribing at the end of life as difficult, examining their ethical considerations, clinical experience, and focus on patient well-being.
